# Machine Learning-Based Classification of ICU-Acquired Neuromuscular Weakness: A Comparative Study in Survivors of Critical Illness

**DOI:** 10.3390/life15121802

**Published:** 2025-11-25

**Authors:** David Estévez-Freire, Ivan Cangas, Andrés Tirado-Espín, Johanna Pozo-Neira, Fernando Villalba-Meneses, Diego Almeida-Galárraga, Omar Alvarado-Cando

**Affiliations:** 1 School of Biological Sciences and Engineering, Universidad Yachay Tech, San Miguel de Urcuqui 100119, Ecuador; luis.estevez@yachaytech.edu.ec (D.E.-F.); ivan.cangas@yachaytech.edu.ec (I.C.); gvillalba@yachaytech.edu.ec (F.V.-M.); 2 School of Mathematical and Computational Sciences, Universidad Yachay Tech, San Miguel de Urcuqui 100119, Ecuador; ctirado@yachaytech.edu.ec; 3 Psychology Brain and Innovation in Neuroscience Group, Universidad Católica de Cuenca, Cuenca 010107, Ecuador; johanna.pozon@ucacue.edu.ec

**Keywords:** ICU-acquired weakness, neurocritical care, atrophy, machine learning, explainable AI, neurorehabilitation

## Abstract

Classifying the severity of intensive-care-unit-acquired muscle atrophy (ICU-AW) is essential for early prognosis and individualized neurorehabilitation, improving functional outcomes in survivors of critical illness. This study evaluated and compared advanced machine learning (ML) algorithms for classifying neuromuscular atrophy in neurocritical patients. Clinical, biochemical, anthropometric, and morphometric data from 198 neuro-ICU patients were retrospectively analyzed. Six supervised ML models—Support Vector Machine (SVM), Multilayer Perceptron (MLP), Extreme Gradient Boosting (XGBoost), TPOT AutoML, AdaBoost, and Multinomial Logistic Regression—were trained using stratified cross-validation, synthetic oversampling, and hyperparameter optimization. Among the most outstanding models, SVM achieved the best performance (accuracy = 93%, ROC-AUC = 0.95), followed by MLP (accuracy = 82.8%, ROC-AUC = 0.93) and XGBoost (accuracy = 80%, ROC-AUC = 0.94). Stability analyses across random seeds confirmed the robustness of SVM and TPOT, with the highest median AUPRC (>0.90). Explainable AI methods (LIME and SHAP) identified BMI, serum albumin, and body surface area as the most influential variables, showing physiologically consistent patterns associated with a classification of muscle loss.

## 1. Introduction

The emergence of muscle atrophy in survivors of critical illness—collectively known as intensive-care-unit-acquired weakness (ICU-AW)—represents a major neurological complication with significant implications for patient recovery, functional outcomes, and quality of life [1]. ICU-AW affects up to 40% of patients on mechanical ventilation and encompasses a spectrum of neuromuscular syndromes triggered by systemic inflammation, immobility, multi-organ dysfunction, and prolonged exposure to sedatives or neuromuscular blocking agents [2]. Within the framework of post-intensive care syndrome, ICU-AW has been consistently associated with delayed weaning from mechanical ventilation, reduced mobility, increased rehospitalization rates, and long-term disability [1,3]. Accurate and early identification of muscle atrophy severity is essential to optimize rehabilitation strategies and predict recovery trajectories [4,5].

The most common way to diagnose these types of diseases is based on different types of evalutions, a common one is the bedside clinical assessment, such as the Medical Research Council (MRC) total score, which can be used in conjunction with other biomedical diagnostic tools such as electrophysiology [6,7]. However, these approaches have practical limitations in the ICU sector. Clinical scores depend on the evaluator and cannot be applied to sedated patients. This limitation poses a challenge for assessment. Although electromyography and nerve conduction studies provide valuable information, they require specialized expertise and are often logistically impractical [8]. Imaging techniques, such as muscle ultrasound, have emerged as non-invasive tools that now present a potential method to quantify muscle thickness and cross-sectional area, offering a dynamic view of muscle wasting [9,10]. Despite this, there is clearly a lack of standardized protocols and a need to validate their predictive capacity in heterogeneous critical populations.

Recent investigations have explored the role of biological markers in the early identification of ICU-AW. Metabolic and nutritional markers (glucose, albumin, prealbumin) have been linked to muscle catabolism and functional decline [11,12]. In patients with cardiogenic shock requiring mechanical circulatory support, biomarkers including creatine kinase and low albumin levels have been proposed as early indicators of ICU-AW [13]. Systematic evaluations identify up to ten interesting biomarker categories, including stress response mediators (GDF-15), neuronal damage markers (neurofilament light chain), and indicators of muscle fiber injury (myoglobin, titin). Several of these biomarker categories show good discriminative accuracy (AUC > 0.80) [14]. Nevertheless, their clinical utility remains limited due to small cohorts, methodological heterogeneity, and lack of large-scale validation. Longitudinal studies further suggest that while biomarker elevations are common in critical illness, they may not consistently discriminate between patients who will or will not develop ICU-AW, underscoring the need for integrative diagnostic frameworks [15].

Artificial Intelligence (AI) based on machine learning (ML) models has been used in recent research, and with the use of such technology, non-linear interactions between different variables can be identified: demographic, clinical, electrophysiological and even imaging [16,17]. This shows how there is a great capacity to identify these patterns with a high precision (AUC) of up to 0.82, which is an improvement compared to common methods [12]. Lei (2025) demonstrated that the combination of muscle ultrasound parameters with inflammatory biomarkers in septic patients generated robust diagnostic nomograms, which allowed the early detection of AW in the ICU [9]. However, the lack of multimodal integration represents a major research gap. No current studies employ ML to fuse clinical, biochemical, anthropometric, and morphometric data for classifying atrophy severity as a diagnostic tool in neurocritical care [18]. Consequently, reproducible, interpretable, and scalable models capable of supporting precision neurorehabilitation remain unavailable.

To address this limitation, it is necessary to conduct a cross-sectional classification analysis using machine learning algorithms to integrate multimodal inputs and objectively categorize the degree of muscle atrophy. This approach seeks to advance early detection and support precision neurorehabilitation in critical care medicine.

For these reasons, this study aims to evaluate and compare the therapeutic utility and diagnostic efficacy of machine learning-based methods for classifying ICU-acquired muscle atrophy in neurocritical patients by integrating clinical variables with quantitative morphometric biomarkers derived from ultrasound. Morphometric characteristics are used as target variables, classified, and analyzed using linear dimensionality reduction methods to capture latent structural patterns associated with atrophy severity. These findings are expected to demonstrate the potential of artificial intelligence in the treatment of neurological complications after critical illness and contribute to the growing field of neurocritical care. In addition, this study offers a comparative analytical approach for assessing the robustness and performance of different reduction strategies in conjunction with artificial oversampling methods to enhance model generalization and data balance. In proving that data-driven dimensionality reduction enhances clinical interpretability and predictive accuracy in the early detection of ICU-acquired weakness, this manuscript is innovative and significant because it introduces a scalable and repeatable machine learning framework specifically tailored to neurocritical care. Its innovation lies in the integration of multimodal data—clinical, biochemical, anthropometric, and morphometric—to construct objective, data-driven atrophy indices; the comparative evaluation of advanced non-linear algorithms under rigorous statistical validation using stratified cross-validation and random-seed robustness testing; and the incorporation of explainable AI techniques to ensure physiological interpretability and clinical transparency. Collectively, these aspects provide a reproducible and generalizable methodological basis that can be extended to broader neurocritical and intensive care contexts.

### Related Work

Recent studies have explored the application of AI and ML in the diagnosis and management of neuromuscular complications in critical care [19,20,21]. Most existing works, however, remain limited to unimodal data sources—focusing on clinical, biochemical, or electrophysiological information independently—without integrating heterogeneous variables into a unified diagnostic model. Lei et al. (2025) [9] developed nomograms combining ultrasound and inflammatory biomarkers for early ICU-AW detection, while Guo et al. (2024) [12] applied supervised ML to clinical datasets with moderate accuracy. Similarly, Song et al. (2025) [14] summarized circulating biomarkers with high discriminative power but lacking multimodal fusion. Despite the valuable insights provided by these studies, none addressed the classification of atrophy severity as a diagnostic tool.

Table 1 summarizes key recent works in the field, highlighting the novelty of the present study, which integrates multimodal variables—clinical, biochemical, anthropometric, and morphometric—through a cross-sectional classification framework designed for reproducibility and clinical interpretability.

## 2. Materials and Methods

**Figure 1:**
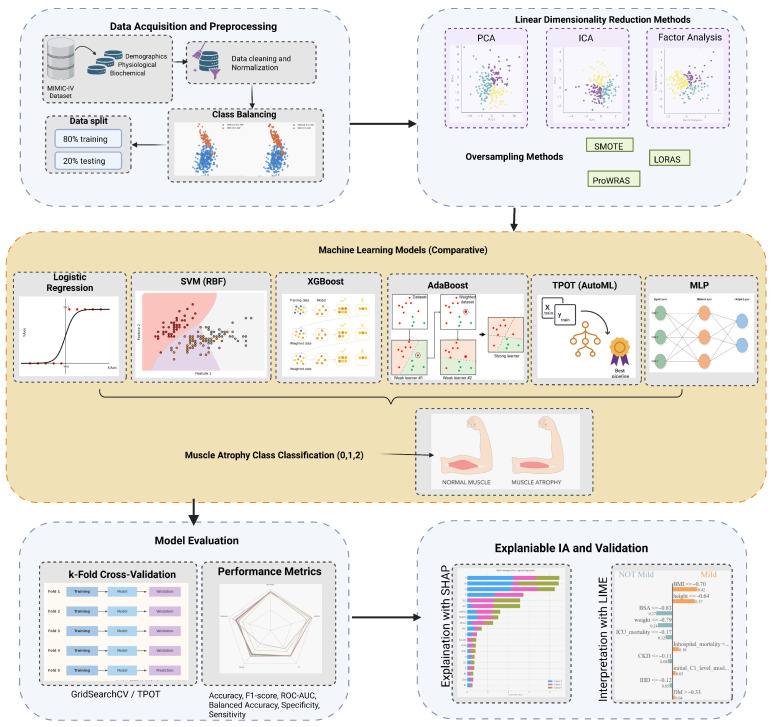
Abstract Methodology.

The methodology presented in this study has reproducibility, scientific validity, and results with clinical relevance, integrating advanced data analysis techniques and class balancing strategies for statistical validation. This section details the data source, cohort selection criteria, preprocessing procedures, implemented algorithms, optimization methods, and procedures to evaluate the interpretability and robustness of the models and their respective results. Figure 1 represents the abstract of the methodology.

### 2.1. Data Description

This is a retrospective, comparative study based on the analysis of the public dataset “Functional Outcomes in ICU Survivors: Muscle and Nerve Function Data” Harvard Dataverse [26]. This resource is one of the most extensive international collections on the neuromuscular and functional outcomes of critically ill people who survived extended ICU admissions. Several clinical, demographic, biochemical, and imaging variables are included, enabling multifactorial modeling of ICU-AW, which is why it is relevant.

The cohort included 198 adult patients who were hospitalized in the neurological ICU between 2010 and 2019 and stayed for at least seven days. The average age was 58.3 years (±14.6), and 52% of the participants were male. Diagnoses included cerebral hemorrhage (22%), ischemic stroke (28%), head trauma (34%), and other serious neurological disorders (16%). Table 2 represents the cohort included in the database.

The dataset included included 53 clinical and morphometric factors. Along with specific morphometric characteristics obtained from cranial tomography and ultrasound studies, these variables also include demographic, anthropometric, biochemical, and outcome-related data. The variables were categorized into continuous and categorical domains (Table 3 and Table 4, respectively) to improve readability and clarity. While categorical variables recorded binary or ordinal clinical conditions like comorbidities, treatment use, and mortality outcomes, continuous variables included quantitative clinical and imaging-derived measurements. The study’s following preprocessing, imputation, and dimensionality reduction analyses were made easier by this organized framework.

**Figure 2:**
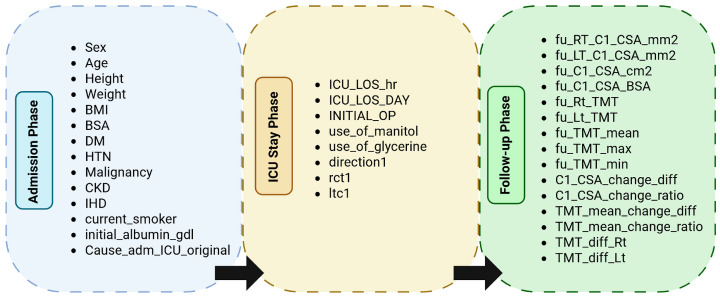
Hierarchical grouping of variables by temporal phase in the dataset. Each phase corresponds to the clinical context of data acquisition: Admission (demographic and baseline measures), ICU Stay (therapeutic and monitoring data), and Follow-up (post-ICU morphometric and outcome variables).

Furthermore, this data was also grouped based on the way it was collected. This allowed us to identify three different categories: the first was Admission, which focuses on basic clinical data of patients before ICU admission. This was followed by data on their ICU stay, and finally, the variables associated with Follow-up, which are comparative data collected from post-ICU patients. This organization facilitated the design of temporal models and clarified the clinical progression structure of the dataset (Figure 2).

The Harvard Dataverse—Functional Outcomes in ICU Survivors: Muscle and Nerve Function Data is a unique resource because it incorporates 3-month functional variables and temporal muscle ultrasound. Early identification necessitates integrating clinical, laboratory, physiology, and imaging data; however, the majority of public databases do not include neuromuscular biomarkers.

The lack of a public standard for neuromuscular imaging/US is the main constraint on the literature; as a result, many earlier ICU-AW models are based on laboratory data and electronic health records, and they simulate atrophy using surrogate variables [27]. By offering validated muscle mass biomarkers in critically ill patients, the dataset from this study closes this gap and makes it possible to analyze and identify targets for machine learning and surrogate evaluation.

The following Table 5 represents a comparison between different public databases in the context of this study.

### 2.2. Data Processing and Preprocessing

Initially, an exploratory analysis of all available variables was performed. A Random Forest analysis was used to obtain the most important variables and thus define our target variable. All continuous predictors were normalized using z scores, and categorical variables were coded using one-hot coding. A comparative evaluation of linear dimensionality reduction methods—Principal Component Analysis (PCA), Independent Component Analysis (ICA), and Factor Analysis—was conducted on the morphometric data to identify the most effective technique for cluster discrimination. Each method was assessed using internal validation indices (Silhouette, Calinski–Harabasz, and Davies–Bouldin) to determine the model with the best balance between compactness, separation, and robustness. K-means clustering was guided by both clinical and statistical criteria. From a biological standpoint, three levels of muscle wasting (no, mild, and moderate atrophy) represent physiologically distinct strata in ICU-acquired weakness studies, aligning with thresholds reported in morphometric ultrasound literature [32]. To validate this decision, an internal silhouette analysis and elbow method were performed on the principal component scores, both showing an inflection point and maximum mean silhouette coefficient

#### Multicollinearity Analysis and Synthetic Oversampling Techniques

A Multicollinearity analysis between variables was assessed using the variance inflation factor (VIF) in order to avoid redundance in clinical variables. In addition, for the initially heterogeneous distribution, applying synthetic oversampling techniques is recommended in the scientific literature [12]. Using SMOTE (Synthetic Minority Over-sampling Technique), LORAS (Localized Random Affine Shadowsampling) and ProWRAS (Proximity Weighted Random Affine Shadowsampling), each method generated new synthetic instances of the minority class, preserving the topological structure of the feature space. Subsequently, the resulting data were reduced to two dimensions using PCA, in order to visualize the generated distributions and evaluate the capacity of each technique to improve the representativeness and homogeneity of the minority samples with respect to the majority class.

One hundred cases per class were generated, achieving a homogeneous dataset that allowed training all the models presented in the study. This procedure was applied only to the training set after the initial partition, avoiding information leakage to the test set.

**Figure 3:**
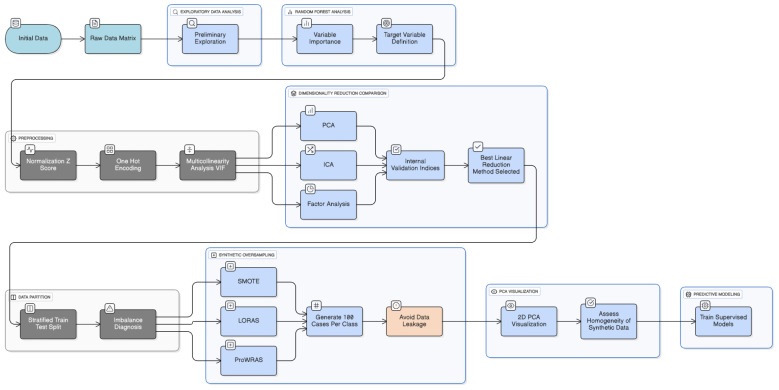
Methodology of preprocessing data.

Finally, different machine learning models were created to classify the degree of atrophy based on the remaining variables, excluding our target variable (categorized using the best linear reduction dimensionality method). Once the target variable is obtained, the predictor variables will be all the others presented in the data set, which contains clinical, demographic, neuromuscular assessment, laboratory data, and medication variables. Figure 3 represents a summary of the preprocessing data in the study.

### 2.3. Machine Learning Models

In this study, several supervised machine learning algorithms were implemented to classify muscle atrophy. All models were trained on the same preprocessed dataset, and hyperparameter optimization was performed using cross-validation.

The SVM, configured with an RBF kernel (C = 1.0, γ = 0.5) and tuned through GridSearchCV, was selected for its capacity to model non-linear relationships in medium-sized, high-dimensional datasets. The TPOT framework automated pipeline design and hyperparameter tweaking through the use of evolutionary optimization using 100 people over 15 generations. Using RandomizedSearchCV (15 iterations, 4-fold CV), XGBoost, an ensemble of gradient-boosted trees, was adjusted to enhance multiclass accuracy by modifying important parameters including tree depth, learning rate, and regularization. As a linear benchmark, MLR with L2 regularization was trained via 4-fold CV and adjusted for stability and convergence. The MLP neural network, comprising one hidden layer of 60 neurons with ReLU activation and Adam optimization, was trained for 60 epochs with L2 regularization ($\alpha \;=\;1\;\times \;10^{-4}$) to capture complex non-linear interactions. Finally, AdaBoost combined 120 shallow decision trees (max_depth = 2) with a learning rate of 0.8 to assess whether sequential boosting could enhance robustness and discrimination relative to gradient-based ensembles.

#### Metrics Evaluated in Machine Learning Models

All models were evaluated in the test set using multiple performance metrics: Precision, record, F1-Score, Matrix confusion, ROC analysis and important variables in the prediction. In the MLP neural network, the learning curves will be presented: train/validation and train/validation loss. The models were trained on a balanced dataset that was randomly partitioned into training (80%) and test (20%) sets using stratified sampling to preserve class proportions. An exception was made for the neural network model, which required a larger validation subset (30%) due to its higher complexity and the need for sufficient data to ensure reliable performance assessment.

In order to evaluate the robustness and reliability of the models, additional analyses were performed by training each algorithm under ten different random seeds. The variability in performance was quantified using seed-dependent metrics such as the area under the precision–recall curve (AUPRC) and the Matthews correlation coefficient (MCC). Furthermore, per-class performance (recall, specificity, precision, balanced accuracy, F2-score) and global indicators (accuracy, MCC, Cohen’s kappa, balanced accuracy, macro-AUPRC, log loss) were calculated. Pairwise statistical comparisons (*p*-values) were also conducted to determine significant differences between models. These results were summarized in tables and boxplots to visualize model stability, calibration, and comparative performance. Finally, model interpretability was assessed using SHAP (SHapley Additive exPlanations) and LIME (Local Interpretable Model-Agnostic Explanations) to validate that the predictions were physiologically coherent and based on clinically relevant variables. SHAP was applied to analyze global feature importance across the entire dataset, identifying the overall contribution and directionality of predictors, while LIME was used to provide local interpretability for individual test samples, illustrating how each feature influenced specific predictions. Together, these explainable AI methods ensured transparency and interpretability, confirming that the decision-making process of the models was both statistically robust and clinically meaningful.

In this context, a summary of model configurations, libraries, and evaluated metrics are represented in Table 6.

**Figure 4:**
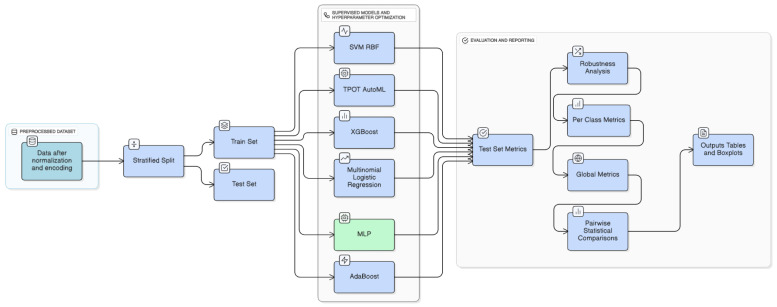
Methodology of machine learning models implemented.

Figure 4 illustrates the overall machine learning workflow and the methodology implemented in this study.

### 2.4. Computational Environment and Hardware Configuration

All analyses were executed on a workstation powered by an AMD Ryzen 7 5800H CPU (3.20 GHz, 8 cores) with 16 GB RAM and a 64-bit operating system. The software environment consisted of Python 3.10, Scikit-learn 1.4.2, Imbalanced-learn 0.12, Pandas 2.2, and Matplotlib 3.8. For model interpretability, SHAP (0.44.1) and LIME (0.2.0.1) were employed, ensuring full reproducibility of both model training and interpretability analyses.

## 3. Results

### 3.1. Data Preprocessing and Validation

Exploratory data analysis revealed that most variables had less than 5% missing values, with the exception of two ultrasound-based measurements (RtTMTmm: 25.8% missing, LtTMTmm: 16.7% missing). Missing values were imputed using k-neighbors, with new data points calculated based on the majority of nearest neighbors in the training set, using a distance metric such as Euclidean.

**Figure 5:**
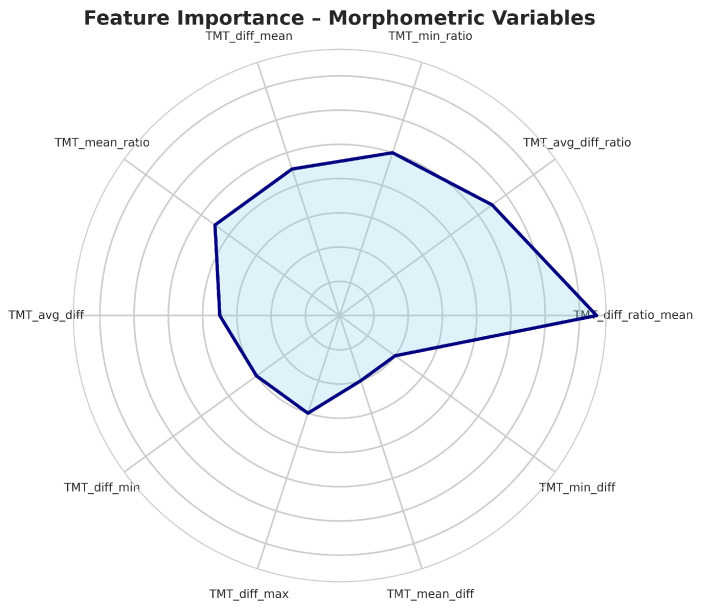
Top most important variables in data set.

Initially, a Random Forest model was developed to obtain the most important variables for classify muscle atrophy in three classes. This is of utmost importance for obtaining and defining our target variable. Figure 5 represents the top 10 most important variables and those that will represent the target of our study.

### 3.2. Target Variables

Feature selection using random forest importance identified the most relevant variables for muscle atrophy classification, primarily morphometric and functional parameters obtained by ultrasound (temporal muscle thickness, cross-sectional area), which were validated as objective biomarkers of muscle atrophy.

**Figure 6:**
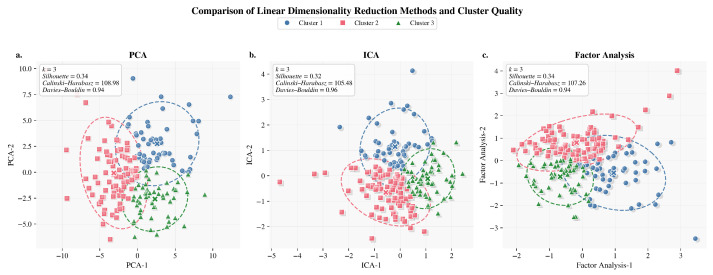
Linear dimensionality reduction comparison methods. (**a**) PCA: projection of three clusters in two principal components. (**b**) ICA: cluster separation in independent component space. (**c**) Factor Analysis: representation of linear separability across factors.

Different linear models for group classification were compared using morphometric variables (see Figure 6), in order to analyze the different parameters and conclude which method to use in the machine learning models.

PCA showed the best overall clustering performance, with a low Davies–Bouldin score (0.94) and the highest Calinski–Harabasz index (108.98), indicating compact, well-defined clusters with little overlap. PCA improved interpretability by offering more stability and variance preservation, even though Factor Analysis produced a comparable Silhouette value. PCA produced more consistent predictions across seeds than ICA, which was more susceptible to noise and correlations. Consequently, PCA is the most reliable and robust technique for evaluating the robustness and stability of clusters in morphometric biomedical data. The following Table 7 represents the metrics described above.

The PCA analysis performed on morphometric variables with the greatest predictive contribution showed that the first five components (PC1, PC2, PC3, PC4, PC5) accounted for 97.5% of the explained variance. The dominance of PC1 demonstrates a high internal correlation between the morphometric indicators, confirming that these variables reflect the same underlying physiological construct: the degree of muscle atrophy. The fact that more than 80% of the total variance is concentrated in the first and second components represents a statistically optimal and biologically consistent result, as it implies that the measurements of temporalis muscle thickness and rates of change respond to a common pattern of structural loss. This behavior validates our data preprocessing and supports the use of PCA as a robust dimensionality reduction technique for morphometric assessment.

From the clustering of PCA scores, the target variable was defined and classified into three categories: no atrophy (*n* = 58), mild atrophy (*n* = 41), and moderate atrophy (*n* = 99).

The principal components derived from the morphometric dataset following dimensionality reduction using PCA were subjected to unsupervised clustering (K-means, k = 3) in order to establish this classification represented in the following Table 8.

This data-driven approach avoided arbitrary clinical cut-offs and ensured that the classification captured natural separations within the morphometric space, as previously recommended in ICU-acquired weakness studies [9].

**Figure 7:**
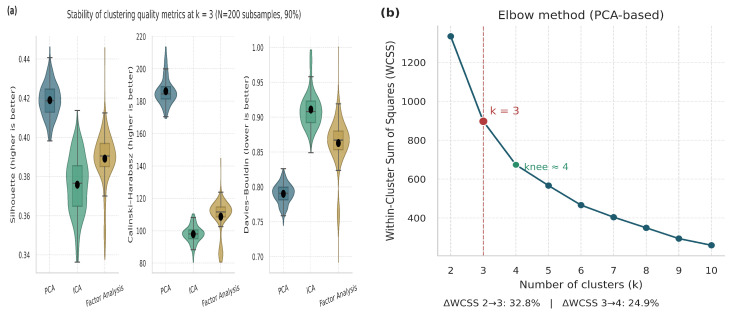
Validation and justification of the selected number of clusters (k = 3). (**a**) Violin and boxplot distributions representing the stability and dispersion of clustering metrics across multiple iterations. (**b**) Elbow plot based on PCA embeddings.

In order to validate k = 3 in PCA, Figure 7 represents the robustness analysis (a) shows consistent Silhouette, Calinski–Harabasz, and Davies–Bouldin scores across 200 subsampling iterations, confirming stability of the three-cluster structure. The elbow plot (b) exhibits the largest reduction in within-cluster variance up to k = 3 (ΔWCSS = 32.8%), after which improvement plateaus. Together, both analyses validate k = 3 as the optimal balance between model simplicity, compactness, and biological interpretability.

### 3.3. Multicollinearity Analysis and Synthetic Oversampling Techniques

**Figure 8:**
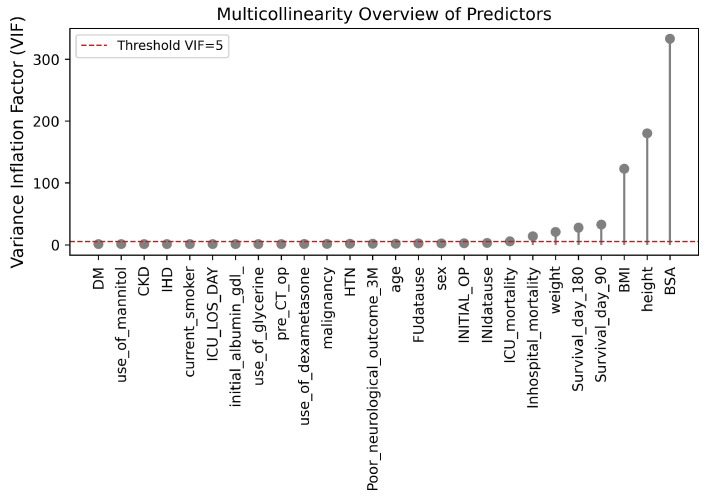
VIF of multicollinearity among predictors.

A multicollinearity study was carried out using the VIF with the objective of analyzing redundancy between clinical variables. Variables related to subsequent outcomes (Survivalday90, Survivalday180) were excluded in order to avoid data leakage and confounding biases associated with treatment. The remaining variables showed acceptable independence (VIF < 5), as represented in Figure 8, except for the anthropometric parameters BMI, height, weight, and BSA, whose VIF values were high, which was expected due to their algebraic dependence. However, they were retained due to their physiological and clinical relevance, as BMI reflects the patient’s nutritional status and BSA allows for normalization of morphometric values according to body size in the ICU [33]. Therefore, this collinearity was considered theoretical and not detrimental to the performance or interpretability of the model.

**Figure 9:**
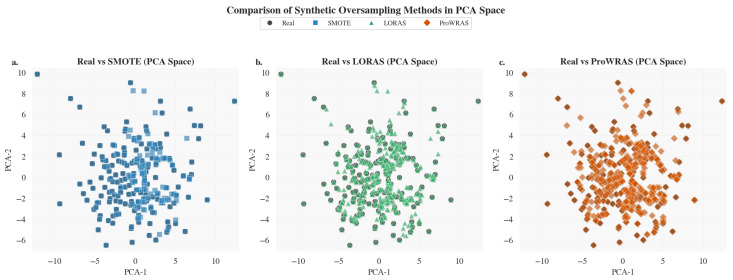
Oversampling methods comparison in PCA space. (**a**) Real vs SMOTE samples visualized in PCA space, showing synthetic data distribution around real instances. (**b**) Real vs LORAS samples demonstrating better boundary diversity and reduced overlap. (**c**) Real vs ProWRAS samples depicting improved class representation and structural preservation.

Finally, it is necessary to perform a comparison of the synthetic oversampling techniques SMOTE, LORAS, and ProWRAS to use the technique with the best results in machine learning models. Figure 9 shows each panel of spatial distribution of real and synthetically generated samples after dimensionality reduction, highlighting differences in data dispersion and cluster density.

The comparative analysis of distributional fidelity metrics demonstrates that SMOTE generated synthetic samples most consistent with the real data distribution, achieving the lowest Kullback–Leibler (KL) divergence and Wasserstein distance across both principal components. These results suggest that SMOTE preserves the intrinsic morphometric variability while avoiding overfitting to minority class regions, a limitation observed in LORAS and ProWRAS (see Table 9). Furthermore, the lower divergence indicates that SMOTE produces a smoother interpolation in feature space, maintaining the underlying linear structure captured by PCA, which enhances the robustness and generalization capacity of subsequent classification models.

### 3.4. Machine Learning Models

#### 3.4.1. SVM Model

**Figure 10:**
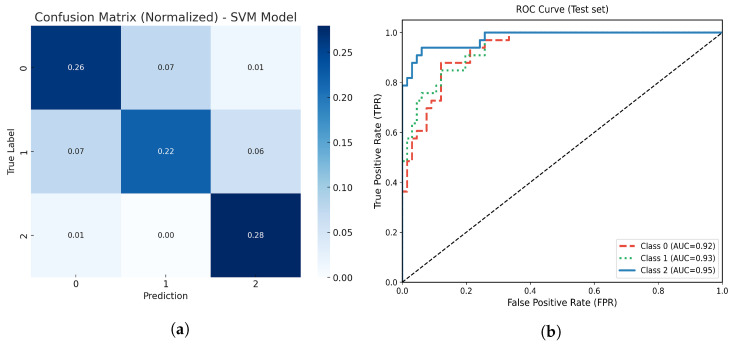
(**a**) Normalized confusion matrix for the optimized SVM model. (**b**) Multiclass ROC-AUC curves for each atrophy class.

The optimized SVM model achieved an overall accuracy of 93%. Figure 10 illustrates the model’s normalized confusion matrix and multiclass ROC-AUC analysis. The SVM demonstrated excellent discriminative performance across all atrophy classes, with several atrophy instances correctly classified. The normalized confusion matrix indicates strong agreement between true and predicted labels, particularly for the moderate atrophy class.

The multiclass ROC analysis yielded an overall AUC of 0.95 for the model, with class-specific values as follows: 0.92 for No Atrophy (class 0), 0.93 for Mild Atrophy (class 1), and 0.97 for Moderate Atrophy (class 2). These results confirm the high sensitivity and specificity of the SVM classifier for detecting varying levels of muscle atrophy.

#### 3.4.2. TPOT AutoML Model

**Figure 11:**
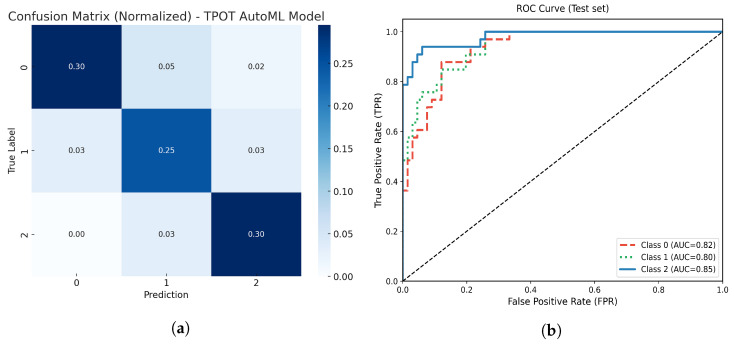
(**a**) Normalized confusion matrix for the optimized TPOT AutoML model. (**b**) Multiclass ROC-AUC curves for each atrophy class.

On the withheld test set, the optimized TPOT AutoML model achieved an overall accuracy of 81%. Figure 11 represents the model’s normalized confusion matrix and multiclass ROC-AUC analysis. The confusion matrix demonstrates high true positive rates for the moderate atrophy class (class 2), with most misclassifications concentrated in the mild atrophy group. This behavior suggests that, while the model effectively detects more severe atrophy, it tends to overlap between mild and moderate categories.

The multiclass ROC analysis yielded AUC values of 0.86, 0.87, and 0.96 for classes 0, 1, and 2, respectively, with an overall macro AUC of 0.89, indicating strong discriminative ability across all classes. These results confirm the robustness of the AutoML-generated pipeline in capturing complex nonlinear relationships among clinical and morphometric features.

#### 3.4.3. Extreme Gradient Boosting

The optimal parameters identified were: maximum depth = X, learning rate = Y, n estimators = Z, subsample = U, colsample = V, regα = W, and regλ = T.

On the withheld test set (20% of the data), the optimized XGBoost model achieved an overall accuracy of 80%.

**Figure 12:**
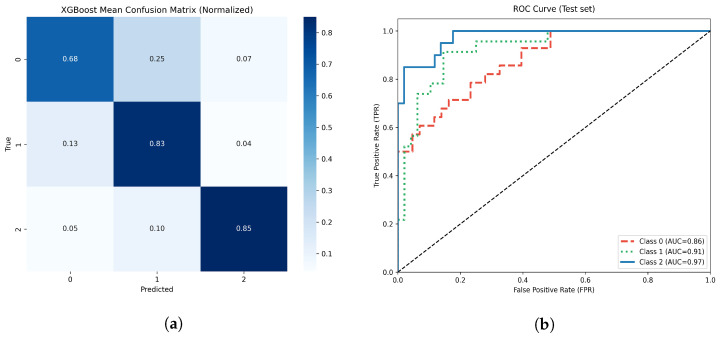
(**a**) Normalized confusion matrix for the optimized XGBoost model. (**b**) Multiclass ROC-AUC curves for each atrophy class.

The normalized confusion matrix (Figure 12a). showed high true positive rates for the no atrophy and moderate atrophy classes, with higher misclassification rates in the mild atrophy group. Multiclass ROC analysis showed AUC values of 0.86, 0.91, and 0.97 for the respective classes (Figure 12b).

The use of SMOTE for class balancing, along with stratified splitting and robust hyperparameter optimization, ensured reliable model generalization to un-observed data.

#### 3.4.4. Logistic Regression

On the independent test set, the optimized logistic regression model achieved an overall accuracy of 79.4%.

**Figure 13:**
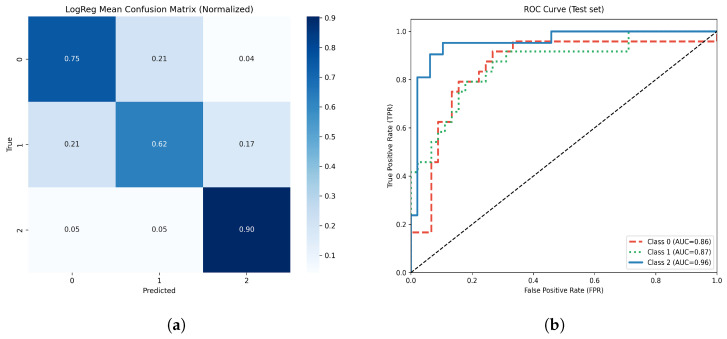
(**a**) Normalized confusion matrix for the optimized Logistic Regression model. (**b**) Multiclass ROC-AUC curves for each atrophy class.

The normalized confusion matrix (Figure 13a) showed the highest true positive rate in the moderate atrophy class, with most misclassifications occurring in the mild atrophy group. The multiclass ROC analysis demonstrated AUC values of 0.86, 0.87, and 0.96 for classes 0, 1, and 2, respectively (Figure 13b). The overall macro ROC-AUC was 0.89.

#### 3.4.5. Neural Network Model—Multilayer Perceptron

MLP neural network was implemented for multiclass classification. The MLP architecture consisted of a single hidden layer with 60 neurons, employing the ReLU activation function and L2 regularization (α = $1\;\times \;10^{-4}$). The network was trained using the Adam optimizer with a learning rate of 0.001, over 60 epochs (maximum iterations per epoch), and with warm restarts enabled incremental learning. At each epoch, the model was updated and both training and validation performance were recorded. To choose the number of epochs, an initial analysis was conducted. Around epoch 60, validation accuracy stabilizes; beyond that, the difference between training and validation starts to grow, which could indicate overfitting. While training on moderate-sized clinical datasets, it is common practice to stop training when it no longer improves validation, as extending beyond that point provides marginal gains and increases the risk of overfitting.

Performance metrics—including accuracy, precision, recall, F1-score, confusion matrices (counts and normalized), learning curves (accuracy and loss), and multiclass ROC-AUC—were calculated on the independent test set. This setup allowed us to monitor model convergence and generalization capability across epochs, as well as to visualize discrimination power for each atrophy group. On the independent test set, the MLP achieved an overall accuracy of 82.8%.

**Figure 14:**
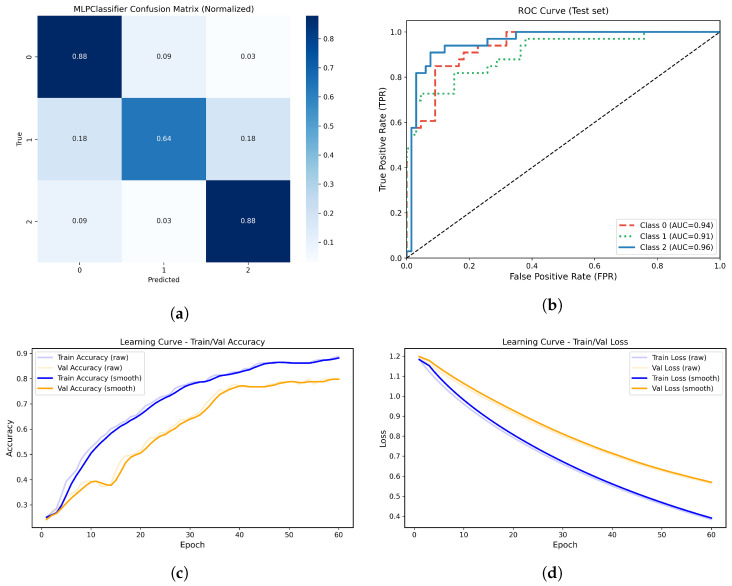
(**a**) Normalized confusion matrix for the MLPClassifier model. (**b**) Multiclass ROC-AUC curves for each atrophy class. (**c**) Learning curve showing training and validation accuracy across epochs. (**d**) Learning curve showing the progressive decrease in training and validation loss across epochs.

The normalized confusion matrix (Figure 14a) demonstrated high true positive rates for the no atrophy and moderate atrophy classes (recall = 0.879 for both), with most misclassifications occurring in the mild atrophy group. The multiclass ROC analysis demonstrated AUC values of 0.94, 0.91, and 0.96 for classes 0, 1, and 2, respectively (Figure 14b).

The learning curves (Figure 14c,d) showed progressive improvement in both training and validation accuracy, as well as a monotonic decrease in loss across epochs, without evidence of significant overfitting.

#### 3.4.6. AdaBoostClassifier

On the independent test set, the optimized AdaBoost model achieved an overall accuracy of 73.7%. Class-specific performance metrics are summarized and illustrated in Table 10. The model showed the highest recall (0.879) and F1 score (0.806) for the moderate atrophy class (class 2), while the mild atrophy group (class 1) exhibited the lowest recall (0.545), indicating a partial overlap with the other classes, that is, consequently, the classification is weak due to the atrophy with class 1, which, despite having a percentage above 0.80 in the other classes, is affected by the other class.

**Figure 15:**
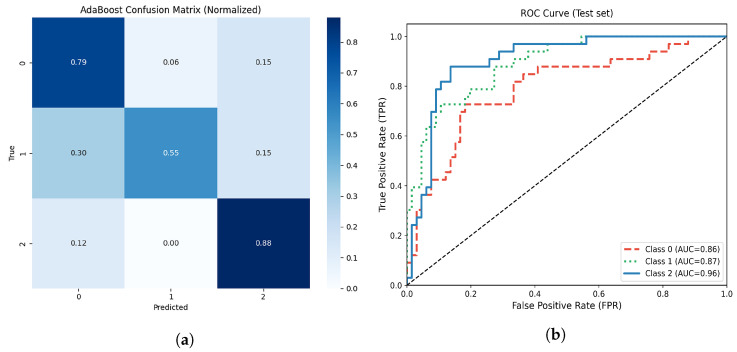
(**a**) Normalized confusion matrix for the optimized AdaBoost model. (**b**) Multiclass ROC-AUC curves for each atrophy class.

Figure 15 represents the confusion matrix of the aforementioned model, AdaBoost, which demonstrates excellent classification performance across the different classes, including those with no atrophy and those with moderate atrophy, with the majority of misclassifications concentrated in the mild atrophy group. The normalized confusion matrix indicates true positive rates of 0.79, 0.55, and 0.88 for classes 0, 1, and 2, respectively.

The multiclass ROC analysis (Figure 15b) yielded AUC values of 0.79, 0.89, and 0.90 for classes 0, 1, and 2, respectively, with an overall macro AUC of approximately 0.86. These results highlight the model’s solid discrimination ability, especially for detecting moderate levels of muscle atrophy.

Although AdaBoost achieved slightly lower overall accuracy than neural network models such as the SVM shown above, it demonstrated balanced recall and robustness to overfitting, confirming its reliability for structured clinical data with limited samples like our data type.

The paremeters described in methodology are represented in Table 10.

### 3.5. Quantitative Comparison of Model Performance

**Figure 16:**
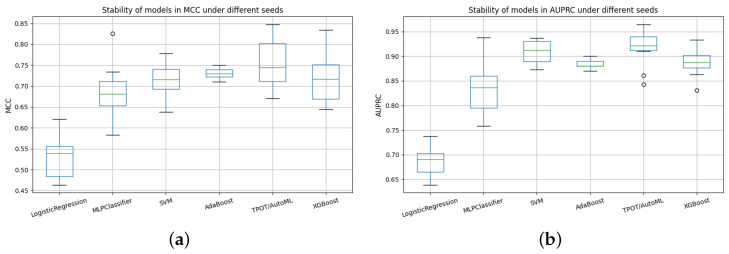
Comparative stability of models across random seeds using AUPRC and MCC metrics. (**a**) Stability of models in AUPRC under different seeds. (**b**) Stability of models in MCC under different seeds.

To comprehensively evaluate the models, their performance was compared across multiple seeds and assessed using metrics specifically suited for multiclass classification. Figure 16 summarizes the stability of the models with respect to the area under the precision–recall curve (AUPRC) and the Matthews correlation coefficient (MCC). Both metrics are particularly relevant in imbalanced scenarios, as they capture discriminatory ability and overall classification quality beyond simple accuracy.

The seed stability analysis based on AUPRC (Figure 16) revealed substantial differences among the models. Logistic Regression consistently exhibited the lowest median AUPRC values (≈0.72) and the highest variability, highlighting its limited capacity to provide stable classifications. MLPClassifier achieved higher median values (∼0.84), but the wide dispersion of results indicated sensitivity to random initialization. In contrast, SVM and TPOT/AutoML achieved the highest median AUPRC values (>0.90) with narrow interquartile ranges, demonstrating superior robustness and reproducibility. XGBoost also achieved competitive values (∼0.89), although with slightly greater variability than SVM.

The MCC-based analysis provided a complementary perspective (also in Figure 16). Logistic Regression again displayed the lowest medians with large variability, confirming its limited reliability in multiclass classification. MLPClassifier and XGBoost attained intermediate medians, although both showed modest dispersion, suggesting moderate robustness. Consistent with the AUPRC analysis, SVM and TPOT/AutoML achieved the highest median MCC values with compact distributions, reinforcing their stability and superior capacity to handle variability across seeds.

In this context the following (Table 11 and Table 12) represents a comparative of models for the MCC and AUPRC values.

In addition, performance at the class level is represented in (Table 13). SVM and TPOT/AutoML achieved the best recall and specificity, particularly in class 2 (moderate atrophy), which is the most clinically relevant group. Logistic Regression showed poor discrimination in class 1 (mild atrophy), while MLPClassifier exhibited more balanced results but with reduced robustness. XGBoost also reached strong values, although its consistency was lower than that of SVM.

Pairwise statistical comparisons (Table 14) confirmed the superiority of non-linear models. Logistic Regression was significantly outperformed by SVM, TPOT/AutoML, and XGBoost (*p*-values < 0.01). No significant differences were found between SVM, TPOT/AutoML, and XGBoost, highlighting their comparable performance. MLPClassifier showed intermediate results, being significantly inferior to TPOT but not to SVM or XGBoost.

Finally, in order to provide a more comprehensive analysis and robust evaluation, a set of extended evaluation metrics were incorporated with this process. These include balanced accuracy, specificity, sensitivity (mean recall), Cohen’s kappa coefficient, MCC, and macro/micro ROC-AUC values. These indicators are important as they complement conventional accuracy and the F1 score in interpreting and assessing model agreement, generalization balance, and robustness to class imbalance.

**Figure 17:**
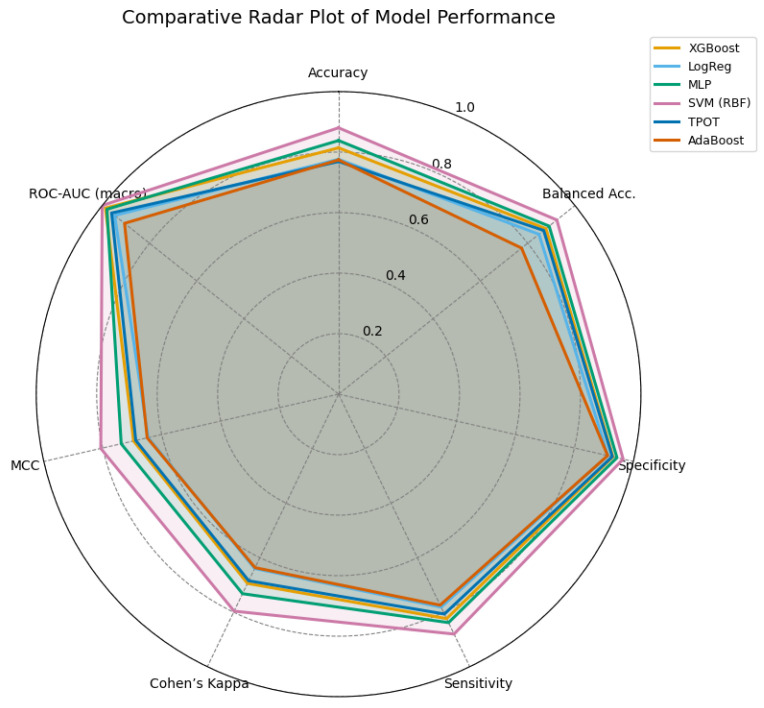
Radar comparison of extended evaluation metrics across all models. A larger enclosed area indicates a more balanced and stable performance. The SVM (RBF) model demonstrates superior overall consistency, followed by the MLP and XGBoost models.

Table 15 illustrates that all models achieved satisfactory performance despite the large number of variables involved, with balanced accuracies above 0.80 and ROC-AUC scores above 0.90. The SVM model outperformed the other models, as can be seen and demonstrated in the rest of the work, obtaining the highest balanced accuracy (0.879), specificity (0.919), and ROC-AUC (0.952), indicating excellent generalization capabilities and strong discrimination across all muscle atrophy classes, suggesting that its classification performance is anything but random. The MLP and XGBoost models closely followed the previous example, showing stable and consistent results with balanced accuracies of 0.848 and 0.835, respectively. In contrast, the models presented, such as logistic regression and TPOT, obtained competitive, albeit slightly lower, scores. This can be attributed to their lower ability to capture nonlinear boundaries, which is equally important for this analysis. Figure 17 provides a graphical summary of the comparative results.

#### Model Interpretability

This section presents the interpretability analysis of the proposed machine learning models using explainable artificial intelligence (XAI) techniques. Both SHAP and LIME were applied, following approaches consistent with recent biomedical studies sharing similar clinical contexts [34].

To generate local feature attributions for representative test samples, interpretability analyses were performed across all models described in the previous sections. Each LIME explainer was trained within the standardized feature space under the same computational framework, defining its corresponding variables. The method was applied to a single representative test observation per model sample0, producing individualized HTML visualizations that illustrate feature contributions to the classified outcome.

**Figure 18:**
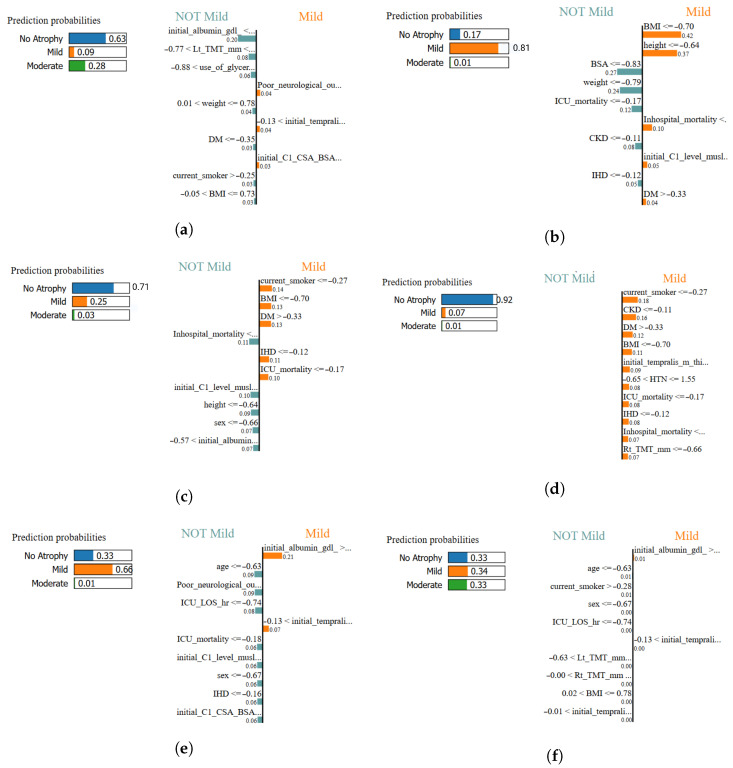
**Local interpretability comparison using LIME across all classifiers.** Each panel illustrates the most influential features contributing positively or negatively to the classification of muscle atrophy class for a representative test sample: (**a**) XGBoost–LIME explanation, (**b**) Logistic Regression–LIME explanation, (**c**) MLPClassifier–LIME explanation, (**d**) SVM (RBF)–LIME explanation, (**e**) TPOT AutoML–LIME explanation, and (**f**) AdaBoost–LIME explanation. In this study, the most influential features highlighted by LIME are primarily associated with anthropometric measures and clinical variables.

LIME analysis provides clear and critical insights into how specific clinical and anthropometric variables influenced individual classifications. The method identifies whether each feature increased (positive weight) or decreased (negative weight) the classification probability of muscle atrophy, thereby supporting the physiological plausibility of the models’ decision-making process. This step was essential to verify that the models not only achieved high accuracy but also made biologically meaningful classifications. Figure 18 summarizes a comparative overview of the top five LIME-derived features across classifiers. Although the exported HTML visualizations were simplified to preserve readability, the consistency in the relative feature magnitude across models demonstrates a shared dependency structure. This indicates that all models rely on a similar subset of dominant features for local decision-making, which aligns with the global interpretability patterns identified using SHAP.

In this study, the most influential features highlighted by LIME are primarily associated with anthropometric measures and clinical variables.

These interpretable results validate the model classifications at the individual level, confirming that the relationships between body composition, disease severity, and muscular deterioration are physiologically coherent. Consequently, the models can be considered both statistically robust and clinically trustworthy.

As shown in Figure 18, LIME explanations consistently highlighted clinically relevant features across all classifiers. Key variables such as BMI, height, albumin concentration and ICU length of stay recurrently influenced classifications, reinforcing the physiological soundness of the models.

Table 16 sumarize the key insights and clinical interpretation of the results presented in Figure 18.

SHAP and LIME provide complementary insights into model interpretability. SHAP captures global feature relevance and consistency across the dataset, while LIME focuses on patient-level reasoning, ensuring that model classification are both accurate and biologically explainable. The convergence of both methods strengthens the transparency and reliability of the proposed machine learning framework for muscle atrophy classification.

## 4. Discussion of Results

### 4.1. Principal Findings and Clinical Implications

Among all evaluated models, SVM achieved the highest overall accuracy (93%) and macro F1 score (0.93), closely followed by MLP (82.8%), TPOT (81%), XGBoost (80%), MLR (79.4%), and AdaBoost (73.7%). The superior performance of SVM may be attributed to its capability to efficiently handle non-linear relationships and small- to medium-sized datasets, which are characteristic of clinical neurocritical care research. SVM demonstrated excellent discrimination across all atrophy categories, particularly for moderate atrophy, highlighting the suitability of radial basis function kernels for modeling complex, multifactorial clinical data.

Clinically, the superior performance of the SVM model presents implications for the management of acquired muscle atrophy in AW-ICU. This model manages to classify with high precision the severity of established atrophy, and the model allows an objective and reproducible stratification of neurocritical patients according to their multimodal profiles. In clinical practice, the assessment of muscle atrophy relies heavily on manual ultrasound interpretation and subjective scales such as the MRC score. The proposed SVM framework addresses these limitations by integrating quantitative ultrasound features with biochemical and anthropometric indicators, generating an automated classification that correlates with both nutritional status and functional prognosis. Thus, patients with moderate atrophy could be prioritized for early mobilization programs, optimized protein-energy supplementation, and closer neurological monitoring, while those with mild atrophy could benefit from preventive protocols. MLP ranked second after SVM, showing stable learning curves and consistent classification performance. Its capacity to capture non-linear feature interactions contributed to robust performance, although its interpretability was slightly reduced compared to SVM. TPOT, despite being a fully automated approach based on evolutionary pipeline optimization, exhibited only a marginal reduction in accuracy relative to MLP and SVM. However, the complexity of its generated pipelines made interpretability more challenging, which can limit its direct clinical applicability.

The pattern is presented through a consensus analysis of the most influential features contributing to multiclass muscle atrophy classification (Table 17), which is particularly pertinent to neurocritical care. The most reliable discriminative features were anthropometric factors like height, weight, BMI, and BSA, underscoring the importance of muscular capital and baseline physiological reserve in neurological patients in severe condition. This result is in line with previous neuro ICU research, which has found that BMI and decreased muscle mass are associated with a higher risk of ICU-AW as well as adverse neurological outcomes [35,36].

Strong classifier values were also demonstrated by biochemical and clinical indicators such as blood albumin levels and hypertension. In neurocritical care cohorts, hypoalbuminemia has been linked to increased morbidity and delayed neurological recovery as a sign of malnutrition and systemic inflammation [2]. Similarly, neuromuscular deterioration may be made worse by chronic hypertension, which is a condition of vascular risk, especially in individuals who have experienced recent cerebral trauma [37]. These results underline the need of thorough hemodynamic and nutritional care in preventing muscle atrophy and promoting the best possible neurological recovery in this population.

Although they were technically consequences of the illness, outcome factors including survival days and poor neurological condition at three months served as indirect indicators of the degree and durability of neuromuscular dysfunction. ICU-AW is a risk factor and a measure of global frailty in neurocritical patients, as evidenced by their strong ability to discriminate between atrophy severity classes in our models, which highlights the reciprocal relationship between muscle health and neurological outcomes [1].

All things considered, our findings support those of earlier multicenter research, highlighting the need of combining anthropometric, clinical, and biochemical factors for early risk assessment of neuromuscular problems in the neuro-ICU. Crucially, conventional clinical indicators remain highly relevant for risk assessment and bedside decision-making despite the development of sophisticated machine learning algorithms. Future studies should concentrate on prospectively verifying these classifications and investigating other modalities, including muscle ultrasonography, to identify at-risk patients even earlier. In the end, early detection may enable focused nutritional and neurorehabilitation plans, which could enhance the quality of life and functional results for individuals who have survived a serious neurological illness.

### 4.2. Quantitative Comparison Across Models

A comprehensive quantitative evaluation was conducted by integrating core and extended performance metrics on the held-out test set (Table 15), together with seed-wise stability analyses using AUPRC and MCC (Figure 16) and per-class operating characteristics (Table 13). Overall, the SVM consistently outperformed the other classifiers, achieving the highest accuracy (0.838), balanced accuracy (0.879), specificity (0.919), and macro/micro ROC–AUC (0.952/0.951), along with top agreement indices (Cohen’s κ = 0.758, MCC = 0.768). The MLP and XGBoost models followed closely, with balanced accuracies of 0.848 and 0.835, respectively, and macro ROC–AUC values above 0.93 and 0.94, confirming strong discriminative capability. TPOT/AutoML and Logistic Regression produced competitive but comparatively lower scores, suggesting limited ability to capture non-linear boundaries in this clinical domain, while AdaBoost achieved intermediate performance with high specificity but modest sensitivity.

These tendencies were validated by seed-based stability studies (Figure 16). SVM and TPOT/AutoML showed resistance to random initialization with narrow interquartile ranges and high medians in both AUPRC and MCC measures. XGBoost had slightly more dispersion but still maintained competitive values. MLP was sensitive to initiation conditions, as evidenced by its considerable variability despite its high medians. Due to its limited reproducibility, logistic regression displayed the widest dispersion and the lowest medians. The seed-level results shown in Table 11 and Table 12 are in agreement with these patterns.

Clinically significant insights were obtained from the class-wise analysis (Table 13). The best recall and specificity combinations for Class 2 (moderate atrophy) were obtained by SVM and TPOT/AutoML (e.g., SVM: TPR = 0.94, TNR = 0.94; TPOT: TPR = 0.94, TNR = 0.88), which is clinically significant for early treatment prioritizing. Additionally, XGBoost demonstrated good precision and very high sensitivity for Class 2 (TPR = 0.97). Class 1 (moderate atrophy), on the other hand, was the hardest to distinguish. AdaBoost had the poorest recall (0.545) and Logistic Regression had the lowest recall (0.48), indicating a physiological overlap between the mild and moderate categories that would call for more cost-sensitive thresholds or informative factors. SVM and MLP both preserved high TPR and balanced accuracy for Class 0 (no atrophy), reducing clinically undesired false positives.

Finally, pairwise statistical comparisons (Table 14) reinforced the superiority of non-linear models. Logistic Regression was significantly outperformed by SVM, TPOT/AutoML, and XGBoost (*p* < 0.01). SVM, TPOT, and XGBoost showed similar top-tier performance with no discernible variations. MLP produced outcomes that were statistically comparable to SVM and XGBoost but significantly worse than TPOT (*p* = 0.002). The SVM model offers the best overall balance between discrimination, stability, and sensitivity–specificity trade-offs, followed by MLP and XGBoost. TPOT provides a reliable automated alternative for investigating competitive model configurations in multiclass clinical classification tasks. These findings are supported by the convergence of global metrics, AUPRC/MCC stability analyses, and per-class behavior.

### 4.3. Model Interpretability (SHAP/LIME) and Biological Plausibility

To ensure clinical transparency and verify that the predictions are physiologically consistent, complemented the algorithm comparison with two explainability techniques: SHAP (global interpretability at the dataset level) and LIME (local interpretability at the instance level). Both were applied to the same standardized feature space and to the five evaluated classifiers (SVM, MLP, XGBoost, logistic regression, Adaboost, and TPOT/AutoML). Figure 18 summarizes the five features with the greatest local contribution per model, while Table 16 summarizes the explanatory scope of each technique.

The overall analysis indicated a stable and consistent pattern across models: anthropometric variables (BMI, height, weight, and especially body surface area), biomarkers (e.g., serum albumin), and clinical severity indicators (e.g., ICU stay) account for a large portion of the total contribution to predicting atrophy class. Imaging morphometric markers—temporal muscle thickness (TMT) and C1 level cross-sectional area (CSA)—repeatedly emerged as dominant features, confirming their value as objective biomarkers of muscle mass in neurocritical patients. This pattern of relevance aligns with previous evidence and the superior performance observed with nonlinear models, particularly SVM.

At the patient level, LIME allowed the prediction to be broken down into positive/negative weights that increase or decrease the probability of belonging to each class (no, mild, or moderate atrophy). In the individual explanations, the same families of variables dominated the decision: BSA/BMI (somatic reserve), albumin (nutritional status/inflammation), ICU stay (severity/clinical course), and TMT/CSA (muscle loss measured by US/CT). Figure 18 shows this convergence between classifiers: despite architectural differences, each model bases its decision on a small, clinically plausible subset of predictors, which strengthens clinical confidence in the system’s outputs.

SHAP–LIME complementarity is key: SHAP captures the overall importance and directionality of variables across the entire set, while LIME verifies that, on a case-by-case basis, decisions are supported by expected physiological mechanisms. Thus, low albumin values and reductions in TMT/CSA contribute positively to the prediction of atrophy, while higher BSA/BMI exerts a protective effect; a prolonged ICU stay acts as a proxy for severity and favors classes with greater atrophy. This agreement between methods, summarized in Table 16, reinforces the clinical tractability of the proposed framework. The explanations provide immediate utility for neurocritical practice: (i) they allow risk stratification based on markers available at the bedside (albumin, anthropometry) and ultrasound biomarkers (TMT/CSA); (ii) they facilitate the objectification of intervention targets (nutritional optimization, early mobilization) when local contributions point to modifiable determinants; and (iii) they help justify decisions before the multidisciplinary team by linking each prediction to a clear physiological narrative.

The most significant variables in model training were mostly associated with the admission or baseline phase of patient care, as indicated by the SHAP and LIME interpretability studies (Figure 17 and Table 16). These include characteristics that are usually available prior to an intensive care unit transfer or the development of acute weakness, such as serum albumin, body mass index, and body surface area. This finding indicates that the multimodal characteristics influencing present classification performance might potentially make excellent candidates for prognostic modeling in the future that aims to predict ICU-acquired muscle atrophy.

To avoid information leakage, temporal outcome variables were treated with caution during preprocessing; when included in local analyses, they should be interpreted as indirect indicators of severity and not as prospective predictors. The consistency between models and seeds described in previous sections, together with the stability of the dominant characteristics observed in Figure 18, suggests that the conclusions do not depend on a specific model, but on robust physiological relationships present in the data.

### 4.4. Comparative with Neurology-Focused Literature

Studies by Chen [38] and Ardila [39] have demonstrated the clinical utility of SVM and XGBoost models for risk prediction in neurocritical care settings. These works underscore the importance of integrating electrophysiological, imaging, and clinical biomarkers for the early diagnosis and prognosis of ICU-AW [40,41]. In parallel, Amarasinghe [42] highlighted that machine learning-based assessment of muscle mass and muscle quality provides a surrogate marker for global neurological status, particularly in patients with traumatic brain injury and stroke.

Our results are highly consistent with these studies. The predictive value of imaging-derived muscle parameters (such as temporal muscle thickness and C1 muscle cross-sectional area), anthropometric indices (BSA, height, BMI), biochemical markers (albumin), and key clinical variables (hypertension, poor 3-month neurological outcome, survival days) supports the growing trend towards precision neurology and personalized rehabilitation protocols in the ICU [39]. The integration of multidimensional predictors enabled our models—particularly the SVM and MLP architectures—to achieve performance metrics comparable or superior to those reported in recent high-impact neurocritical care literature.

In order to contextualize the contribution of this framework, Table 18 summarizes the most relevant studies found in the literature that apply machine learning to assess muscle atrophy or acquired weakness. Previous research can be divided into two methodological categories: predictive models, which seek to estimate the risk or onset of muscle weakness in the ICU before it manifests, and classification models, which seek to quantify or grade the severity of established atrophy. While predictive approaches support prevention and early detection, severity grading provides an equally important diagnostic function: it offers an objective, data-driven stratification of neuromuscular impairment that can directly inform rehabilitation prioritization and clinical decision-making.

These findings support the use of AI-driven methods in neurocritical care, which integrate multimodal data to predict functional recovery trajectories, facilitate accurate risk classification, and guide early neurorehabilitation. The potential of nonlinear and neural network-based techniques for complex neurological prognostication is evidenced by the superior performance of SVM and MLP models compared to conventional methods. Although the risk of overfitting is reduced by our thorough cross-validation, limitations include single-center data and no external validation. Future research should incorporate longitudinal outcome assessment and other neurological biomarkers (such as electromyography or electroencephalography, and neuroimaging).

### 4.5. Limitations and Future Work

The present study are limited by its sample size (*n* = 198), which is relatively small for training high-dimensional or complex machine learning architectures, such as multilayer perceptrons. While rigorous cross-validation, stratified sampling, and synthetic oversampling were implemented to mitigate overfitting, the performance of the deep models remains limited by data availability.

Additionally, another limitation of the present study is its reliance on a single data source, which may introduce dataset-specific bias and limit the generalizability and interpretability of the results. Future work should aim to validate the proposed framework using independent and heterogeneous databases to assess the model’s robustness and stability across different data distributions.

Although the database includes a diverse set of clinical variables that enhance model performance, the number of patients remains limited. Future studies are encouraged to validate the proposed models in larger cohorts of at least *n* = 500 patients. Furthermore, per-class performance revealed that mild atrophy (class 1) remains the most challenging to discriminate, as reflected by lower recall in linear models and moderate improvement in non-linear ones. Addressing this limitation may require implementing cost-sensitive learning or class-specific thresholding strategies.

In order to create prediction models based on the most significant baseline variables found in this study, future research should make use of the insights from the SHAP and LIME studies. Since a large proportion of these features correspond to the admission phase—prior to the onset of overt muscle weakness—they represent valuable candidates for forecasting the risk and trajectory of ICU-acquired atrophy. Building predictive frameworks on these early indicators could enable proactive intervention and optimized rehabilitation planning in neurocritical care.

Finally, future research should focus on validating these findings in larger, multicentric, and prospective cohorts to ensure generalizability and clinical applicability. The integration of such machine learning pipelines into real-time ICU systems could enable early identification of high-risk patients, support personalized rehabilitation strategies, and ultimately improve neuromuscular outcomes in critically ill individuals.

## 5. Conclusions

The strengths of this study lie in the integration of multidimensional clinical, anthropometric, biochemical, and morphometric data from neurocritical patients, together with the systematic evaluation of multiple supervised learning algorithms and interpretability frameworks. This comprehensive analytical approach allowed the identification of robust and physiologically meaningful predictors of ICU-acquired muscle atrophy and demonstrated the clinical potential of artificial intelligence tools in neurocritical care research.

The results illustrates that machine learning models, particularly SVM and MLP, provide reliable and a precise classification of the degree of neuromuscular atrophy, significantly outperforming conventional regression-based methods and clinical evaluation alone. In addition, SVM with an RBF kernel produced the most reliable and consistent results across all evaluation measures, based on a comparative analysis of six supervised machine learning models. SVM and TPOT/AutoML demonstrated excellent repeatability and robustness to random initialization, maintaining the highest median scores (>0.90) with the lowest variability, based on AUPRC- and MCC-based seed stability assessments. In contrast, MLP and XGBoost performed medium–highly but showed greater inter-seed variance, while Logistic Regression exhibited wide spread and low discriminative power.

Extended evaluation metrics further reinforced these findings: the SVM model achieved the highest balanced accuracy (0.879), specificity (0.919), and macro/micro ROC-AUC (≈0.95), confirming its superior generalization ability. MLP and XGBoost followed closely, also exhibiting strong discriminative power (ROC-AUC > 0.93). From a clinical perspective, the use of such a multimodal and explicable framework may give medical professionals an objective way to grade neuromuscular damage, assisting with rehabilitation triage, personalized treatment planning, and estimating prognoses for patients in critical condition. This is in line with ongoing initiatives to incorporate data-driven diagnostics into ICU decision-support systems and precision neurorehabilitation.

Nonetheless, these findings should be interpreted in light of the study’s single-center, retrospective design and moderate sample size (*n* = 198), which may limit external generalizability and the scalability of more complex deep learning models. Future multicenter and prospective validations will be required to confirm reproducibility across diverse ICU populations.

The SHAP and LIME analyses validated that model decisions were physiologically consistent and driven by relevant variables such as serum albumin, body mass index, height, body surface area, survival time, and neurological outcome. These findings highlight the importance of integrating diverse patient information for a more precise risk stratification and for improving clinical decision-making in ICU settings. Robustness analyses across multiple random seeds and per-class metrics reinforced the superiority of non-linear models for capturing complex pathophysiological relationships, particularly in the identification of mild atrophy cases. SVM showed the highest stability, calibration, and discriminative power, followed by MLP and TPOT/AutoML, while logistic regression exhibited the expected limitations of linear models in high-dimensional, non-linear clinical contexts.

Building on these findings, future studies should use prospective prediction frameworks, externally validated in separate, multicenter cohorts, and use baseline admission data to forecast the onset of atrophy at day 7 or 14. These developments may make it possible to develop hybrid models that combine diagnostic and predictive capabilities, improving precision medicine in neurocritical care.

## Figures and Tables

**Table 1:** Summary of selected research articles on the application of machine learning in intensive care and neuromuscular rehabilitation.

Ref	Description	Type of Study	Use ML/AI	Data Modality
[9]	Combined ultrasound and inflammatory biomarkers for early ICU-AW detection.	Experimental	Yes	Bimodal (Ultrasound + Biomarkers)
[12]	Used supervised ML to predict ICU-AW from clinical and biochemical data.	Retrospective cohort	Yes	Unimodal (Clinical)
[22]	Compared multiple circulating biomarkers for ICU-AW diagnosis.	Systematic review	Partial	Unimodal (Biochemical)
[23]	Applied CNNs on electrophysiological data to assess neuromuscular activity.	Experimental	Yes	Unimodal (Electrophysiology)
[2]	Evaluated clinical predictors of ICU-AW without ML integration.	Observational	No	Clinical data
[24]	Reviewed AI applications in neuromuscular disorder diagnosis and therapy.	Literature review	Yes	Neuromuscular datasets
[25]	Described AI and robotics in physiotherapy and neurorehabilitation.	Review	Yes	Physiotherapy data
**This study**	Integrates multimodal clinical, biochemical, and morphometric data for ICU-AW severity classification.	Cross-sectional classification	**Yes**	**Multimodal (Clinical + Biochemical + Morphometric)**

**Table 2:** Main demographic and clinical characteristics of the cohort (*n* = 198).

Variable	Mean ± SD/*n* (%)
Age (years)	58.3 ± 14.6
Male sex	103 (52%)
ICU length of stay (days)	17.9 ± 6.4
Mechanical ventilation	142 (71.7%)
Hypertension	95 (48%)
Diabetes mellitus	64 (32%)
Serum albumin (g/dL)	3.1 ± 0.6
Body surface area (m^2^)	1.78 ± 0.23

**Table 3:** Continuous variables included in the analysis (*n* = 198).

Variable	Description	Unit
age	Age at ICU admission	years
height	Patient height	cm
weight	Patient weight	kg
BMI	Body Mass Index	kg/m^2^
BSA	Body Surface Area	m^2^
initial_albumin_gdl_	Initial serum albumin	g/dL
initial_C1_level_muslce_CSA	Initial C1 muscle cross-sectional area	mm^2^
initial_C1_CSA_BSA	Initial C1 CSA normalized by BSA	mm^2^/m^2^
Rt_TMT_mm	Right temporalis muscle thickness	mm
Lt_TMT_mm	Left temporalis muscle thickness	mm
initial_tempralis_m_thickness_mean	Initial temporalis mean thickness	mm
initial_tempralis_m_thickness_max	Initial temporalis maximum thickness	mm
initial_tempralis_m_thickness_min	Initial temporalis minimum thickness	mm
fu_Rt_TMT	Follow-up right TMT	mm
fu_Lt_TMT	Follow-up left TMT	mm
fu_TMT_mean	Follow-up mean TMT	mm
fu_TMT_max	Follow-up maximum TMT	mm
fu_TMT_min	Follow-up minimum TMT	mm
fu_C1_CSA_BSA	Follow-up C1 CSA normalized by BSA	cm^2^/m^2^
C1_CSA_change_diff	Absolute change in C1 CSA	mm^2^
C1_CSA_BSA_change_ratio	Relative change in C1 CSA/BSA	ratio
TMT_max_change_diff	Absolute change in TMT (max)	mm
TMT_mean_change_diff	Mean TMT change difference	mm
TMT_min_change_diff	Minimum TMT change difference	mm
TMT_diff_Rt	Right TMT difference	mm
TMT_diff_Lt	Left TMT difference	mm
TMT_diff_mean	Mean TMT difference	mm
TMT_diff_ratio_mean	Mean TMT ratio difference	ratio
TMT_diff_ratio_max	Maximum TMT ratio difference	ratio
TMT_diff_ratio_min	Minimum TMT ratio difference	ratio
TMT_mean_change_ratio	Average change ratio in TMT	ratio
TMT_min_change_ratio	Minimum change ratio in TMT	ratio
TMT_average_change_diff	Average absolute TMT change	mm
TMT_average_change_diff_ratio	Average relative TMT change ratio	ratio
ICU_LOS_DAY	ICU length of stay	days
Survival_day_90	Survival time at 90 days	days
Survival_day_180	Survival time at 180 days	days

**Table 4:** Categorical and binary variables included in the analysis (*n* = 198).

Variable	Description	Unit
sex	Biological sex (0 = Female, 1 = Male)	-
DM	Diabetes mellitus (0 = No, 1 = Yes)	-
HTN	Hypertension (0 = No, 1 = Yes)	-
malignancy	Any malignancy (0 = No, 1 = Yes)	-
CKD	Chronic kidney disease (0 = No, 1 = Yes)	-
IHD	Ischemic heart disease (0 = No, 1 = Yes)	-
current_smoker	Current smoker (0 = No, 1 = Yes)	-
INITIAL_OP	Initial operative management (0/1)	-
pre_CT_op	Pre-CT operation (0/1)	-
ICU_mortality	ICU mortality (0/1)	-
Inhospital_mortality	In-hospital mortality (0/1)	-
GOS_3M	Glasgow Outcome Scale at 3 months (ordinal 1–5)	ordinal
Poor_neurological_outcome_3M	Poor neurological outcome (GOS ≤ 3)	-
use_of_mannitol	Use of mannitol (0/1)	-
use_of_glycerine	Use of glycerine (0/1)	-
use_of_dexametasone	Use of dexamethasone (0/1)	-

**Table 5:** Comparative landscape of public ICU datasets for ML on neuromuscular outcomes.

Dataset	Size	EHR/Labs	High-Res Vitals	Imaging	Neurom. Data	Functional Outcomes	Ref.
Harvard DVN (this study)	*n* = 198, Neuro-ICU	Yes	–	Yes (US-based)	Yes (TMT/CSA, MRC)	Yes (3-months)	[26]
MIMIC-III	∼60k ICU stays	Yes	Yes	–	–	–	[28]
eICU-CRD	Multi-center US	Yes	Yes	–	–	–	[29]
HiRID	Single-center, high-res	Yes	Yes (1-min)	–	–	–	[30]
UMCdb	Multi-center EU	Yes	Yes	–	–	–	[31]

**Table 6:** Configuration and optimization summary of supervised machine learning models for muscle atrophy classification.

Model (Library)	Configuration and Hyperparameters	Optimization Strategy
SVM (scikit-learn)	RBF kernel (C = 1.0, γ = 0.5); suitable for moderate-sized, high-dimensional data with non-linear relationships.	GridSearchCV (5-fold cross-validation).
TPOT AutoML (tpot)	Genetic programming-based AutoML; population = 100, generations = 15; automatic pipeline exploration and early stopping.	Evolutionary optimization with internal cross-validation.
XGBoost (xgboost)	Gradient-boosted decision trees; tuned parameters: max_depth, learning_rate, n_estimators, subsample, colsample_bytree, reg_alpha, reg_lambda.	RandomizedSearchCV (15 iterations, 4-fold CV).
Multinomial Logistic Regression (scikit-learn)	Baseline linear model with L2 regularization; solvers: lbfgs, saga, newton-cg; maximum 500 iterations.	GridSearchCV (4-fold CV) to optimize *C* and ensure convergence.
MLP (scikit-learn)	Single hidden layer (60 neurons), ReLU activation, Adam optimizer, learning rate = 0.001, L2 regularization ($\alpha \;=\;1\;\times \;10^{-4}$); trained for 60 epochs.	Learning curves monitored for training and validation accuracy/loss.
AdaBoost (scikit-learn)	Base estimator: DecisionTreeClassifier (max_depth = 2); 120 estimators, learning rate = 0.8, random state = 42.	Direct evaluation with consistent preprocessing across models.
Evaluation Metrics (common to all models)	Global: Accuracy, Macro F1, ROC–AUC, AUPRC, Log Loss, MCC, Cohen’s Kappa. Per–class: Recall, Precision, Specificity, Balanced Accuracy, F2–Score. Robustness: 10 random seeds with seed-dependent AUPRC and MCC variability. Interpretability: SHAP (global) and LIME (local) analyses for explainability.	Applied consistently across all algorithms for performance benchmarking and validation stability.

All models were implemented in Python using scikit-learn.

**Table 7:** Comparative clustering quality metrics across linear dimensionality reduction methods.

Method	Silhouette	Calinski–Harabasz	Davies–Bouldin
PCA	0.34	108.98	0.94
ICA	0.32	105.48	0.96
Factor Analysis	0.34	107.26	0.94

**Table 8:** Data-driven classification of muscle atrophy levels based on morphometric parameters (TMT and CSA).

Atrophy Level	Definition Criteria
No atrophy	Patients with preserved temporalis muscle thickness (TMT) and C1 cross-sectional area (CSA) within ±1 standard deviation of the cohort mean.
Mild atrophy	Individuals showing moderate reductions in either TMT or CSA between 1–2 standard deviations below the cohort mean.
Moderate atrophy	Patients with severe reductions (>2 standard deviations below the mean) in both TMT and CSA, indicating significant muscle wasting.

**Table 9:** Comparative distributional fidelity metrics for oversampling algorithms in PCA-reduced morphometric space. Lower values indicate higher similarity between real and synthetic data distributions.

Method	KL_PC1_	WD_PC1_	KL_PC2_	WD_PC2_
SMOTE	0.0302	0.5712	0.0227	0.0849
LORAS	0.0667	0.6900	0.0412	0.2007
ProWRAS	0.0631	0.7244	0.0551	0.2382

**Table 10:** Comparative performance of six supervised ML models for muscle atrophy classification.

Condition	Metric	SVM (RBF)	TPOT/AutoML	XGBoost	Logistic Reg.	MLPClassifier	AdaBoost
	Precision	0.920	0.820	0.826	0.850	0.763	0.650
No Atrophy (0)	Recall	0.910	0.790	0.679	0.762	0.879	0.788
	F1-score	0.915	0.805	0.745	0.750	0.817	0.716
	Precision	0.930	0.800	0.701	0.714	0.840	0.690
Mild (1)	Recall	0.920	0.780	0.826	0.625	0.736	0.545
	F1-score	0.915	0.790	0.745	0.667	0.724	0.679
	Precision	0.950	0.850	0.920	0.792	0.806	0.744
Moderate (2)	Recall	0.930	0.830	0.850	0.905	0.879	0.879
	F1-score	0.940	0.840	0.894	0.844	0.841	0.806
	Mean F1	0.928	0.810	0.795	0.754	0.794	0.732
Overall	Weighted F1	0.931	0.820	0.795	0.759	0.801	0.732
	Accuracy	0.930	0.810	0.800	0.794	0.828	0.737

**Table 11:** AUPRC results by seed for the different classification models.

Seed	Logistic Regression	MLPClassifier	SVM	TPOT/AutoML	XGBoost	AdaBoost
0	0.6821	0.8031	0.8539	0.9070	0.9116	0.8217
1	0.7458	0.8462	0.8816	0.8941	0.8396	0.7635
2	0.8187	0.9569	0.9748	0.9809	0.8990	0.8641
3	0.7112	0.8146	0.9318	0.9105	0.8939	0.7916
4	0.6929	0.8261	0.8646	0.8876	0.8804	0.8196
5	0.6828	0.8329	0.8487	0.8959	0.8571	0.7447
6	0.7251	0.8130	0.8853	0.8952	0.8883	0.9101
7	0.7690	0.8719	0.9049	0.9118	0.8565	0.8079
8	0.7475	0.8798	0.9158	0.9165	0.9127	0.8262
9	0.6768	0.8216	0.9071	0.9384	0.8918	0.8196

**Table 12:** MCC results by seed for the different classification models.

Seed	Logistic Regression	MLPClassifier	SVM	TPOT/AutoML	XGBoost	AdaBoost
0	0.470	0.595	0.683	0.832	0.743	0.6674
1	0.549	0.639	0.713	0.681	0.782	0.5477
2	0.625	0.865	0.835	0.879	0.911	0.7446
3	0.427	0.752	0.688	0.745	0.623	0.6095
4	0.471	0.636	0.698	0.668	0.622	0.6086
5	0.364	0.668	0.648	0.780	0.668	0.5191
6	0.583	0.805	0.758	0.789	0.716	0.7605
7	0.534	0.731	0.776	0.655	0.654	0.6522
8	0.501	0.743	0.691	0.758	0.728	0.6456
9	0.471	0.643	0.732	0.774	0.743	0.6225

**Table 13:** Per-class metrics for the different models, including AdaBoost.

Model	Class	Recall (TPR)	Specificity (TNR)	Precision	Balanced Acc	F2 Score	Support
XGBoost	0	0.73	0.86	0.73	0.80	0.73	33
	1	0.61	0.91	0.77	0.76	0.63	33
	2	0.97	0.88	0.80	0.92	0.93	33
Logistic Regression	0	0.73	0.82	0.67	0.77	0.71	33
	1	0.48	0.83	0.59	0.66	0.50	33
	2	0.82	0.86	0.75	0.84	0.80	33
MLPClassifier	0	0.88	0.86	0.76	0.87	0.85	33
	1	0.64	0.94	0.84	0.79	0.67	33
	2	0.88	0.89	0.81	0.89	0.86	33
SVM (RBF)	0	0.91	0.83	0.73	0.87	0.87	33
	1	0.67	0.98	0.96	0.83	0.71	33
	2	0.94	0.94	0.89	0.94	0.93	33
TPOT/AutoML	0	0.70	0.86	0.72	0.78	0.70	33
	1	0.70	0.92	0.82	0.81	0.72	33
	2	0.94	0.88	0.79	0.91	0.91	33
AdaBoost	0	0.76	0.82	0.68	0.79	0.74	33
	1	0.70	0.89	0.77	0.80	0.71	33
	2	0.79	0.91	0.81	0.85	0.79	33

**Table 14:** Pairwise statistical comparisons between models (*p*-values).

Comparison	*p*-Value	Interpretation
Logistic Regression vs. MLP	0.436	Not significant
Logistic Regression vs. SVM	0.002	Significant (SVM better)
Logistic Regression vs. TPOT	<0.001	Highly significant (TPOT better)
Logistic Regression vs. XGB	0.006	Significant (XGBoost better)
MLP vs. SVM	0.276	Not significant
MLP vs. TPOT	0.002	Significant (TPOT better)
MLP vs. XGB	0.436	Not significant
SVM vs. TPOT	0.436	Not significant
SVM vs. XGB	≈0.999	Not significant
TPOT vs. XGB	0.276	Not significant

**Table 15:** Performance metrics of the evaluated machine learning models, including AdaBoost.

(a) Core Performance Metrics				
Model	Accuracy	Balanced Acc.	Specificity	Sensitivity
XGBoost	0.80	0.835	0.886	0.784
LogReg	0.794	0.807	0.870	0.744
MLP	0.828	0.848	0.899	0.798
SVM (RBF)	0.93	0.879	0.919	0.838
TPOT	0.81	0.826	0.884	0.768
AdaBoost	0.737	0.737	0.869	0.737
**(b) Extended Evaluation Metrics**				
**Model**	**Cohen’s κ**	**MCC**	**ROC-AUC (Macro)**	**ROC-AUC (Micro)**
XGBoost	0.661	0.662	0.939	0.939
LogReg	0.609	0.615	0.902	0.904
MLP	0.697	0.702	0.933	0.931
SVM (RBF)	0.758	0.768	0.952	0.951
TPOT	0.652	0.655	0.914	0.914
AdaBoost	0.606	0.618	0.862	0.856

All metrics were averaged across 10 random seeds with stratified 5-fold cross-validation. The SVM (RBF) achieved the highest overall performance across all evaluation criteria.

**Table 16:** Interpretation summary of the XAI methods applied in this study. SHAP provides global interpretability across the dataset, while LIME offers local interpretability for individual classifications, ensuring both analytical and clinical transparency.

Method	Explanation Scope	Key Insights	Clinical Interpretation
SHAP	Global (dataset-level)	Determines the overall contribution and directionality of features across all classifications.	Identifies BMI, BSA, ICU stay duration as dominant classifiers of muscle atrophy.
LIME	Local (instance-level)	Provides individualized explanations showing how specific variables influenced a single classification.	Confirms that patient-level decisions follow physiologically coherent patterns, supporting clinical trustworthiness.

**Table 17:** Top consensus features for multiclass muscle atrophy classification based on integrated mean importance across all machine learning models.

Feature	Variable Type	Integrated Mean Importance
BSA (Body Surface Area)	Anthropometric	0.72
Height	Anthropometric	0.71
Temporal Muscle Thickness (min)	Imaging	0.69
HTN (Hypertension)	Clinical	0.54
Poor Neuro Outcome (3M)	Outcome	0.63
Albumin	Biochemical	0.49
C1 Muscle CSA	Imaging	0.42
BMI (Body Mass Index)	Anthropometric	0.52
Survival days	Outcome	0.36
Weight	Anthropometric	0.38

**Table 18:** Comparative overview of machine learning studies addressing muscle atrophy in critical and neurocritical care, distinguishing predictive and classification approaches.

Reference	Cohort/Setting	ML Model(s)	Primary Task	Performance (Acc/AUC)
**I. Predictive Models (ICU-AW Onset/Risk Prediction)**				
[38]	NeuroICU (*n* = 120, TBI/stroke)	SVM, Random Forest	Predict ICU-AW onset using electrophysiology, TMT, BMI, albumin	AUC ≈ 0.92 (SVM)
[39]	Multicenter ICU (*n* = 210)	XGBoost, Logistic Regression	Predict ICU-AW risk from ultrasound, SOFA, hypertension	Acc. ≈ 0.85 (XGB)
[12]	ICU (sepsis subgroup, *n* = 184)	LR, RF, XGB (+LASSO)	Predict ICU-AW from cytokines, APACHE II, albumin	Acc. ≈ 0.88
[43]	ICU (MV ≥ 7d, *n* = 97)	LASSO, RF, LR	Predict poor mobility outcome from SAPS 3 + Perme scale	Acc. ≈ 0.80
[37]	ICU (prospective, *n* = 749)	XGBoost, RF, SVM, LR	Predict ICU-AW from multimodal clinical + lab + US data	Acc. = 0.978 (XGB)
**II. Classification Models (Atrophy Severity/Neuromuscular Grading)**				
[9]	Septic ICU (*n* = 128)	Random Forest, Logistic Regression	Classify three-grade ICU-AW severity (MRC-based)	AUC = 0.83 (RF)
[42]	Sarcopenia (*n* = 40)	CNN U-Net	Classify binary (CT muscle area)	Acc. = 0.96
[44]	Neuro-oncology (GBM)	CNN (Deep Learning)	Classify temporalis atrophy index (CSA segmentation)	AUC = 0.84
[45]	MRI neuro dataset (*n* = 23,876)	DL pipeline (iTMT)	Automated grading of TMT (percentile-based)	AUC = 0.90
[46]	Acute ischemic stroke (*n* = 264)	Deep CNN (MRI)	Dichotomous classification: severe vs. non-severe TMT loss	AUC = 0.85
**Present study**	NeuroICU (*n* = 198)	**SVM, XGB, MLP, AdaBoost, TPOT, Logistic**	**Three-class atrophy severity (no, mild, moderate)**	**Acc. = 0.93; AUC = 0.95 (SVM)**

## Data Availability

The dataset used in this study is openly available at https://dataverse.harvard.edu/dataset.xhtml?persistentId=doi:10.7910/DVN/GF08RY (accessed on 1 September 2025).
